# A genome-wide interaction study of thyroid-stimulating hormone levels and particulate matter exposure among Koreans

**DOI:** 10.1186/s41021-026-00357-z

**Published:** 2026-03-05

**Authors:** Young Jun Park, Hyun-Jin Kim, Ho-Young Son, Juhyun Kim, Jae Moon Yun, Hyuktae Kwon, Belong Cho, Jin-Ho Park, Jong-Il Kim

**Affiliations:** 1 Genomic Medicine Institute, Medical Research Center, Seoul National University, Seoul, Republic of Korea; 2 Department of Family Medicine, Seoul National University Hospital, Seoul, Republic of Korea; 3 National Cancer Control Institute, National Cancer Center, Goyang, Republic of Korea; 4 Department of Family Medicine, Seoul National University College of Medicine, Seoul, Republic of Korea; 5 Institute on Aging, Seoul National University College of Medicine, Seoul, Republic of Korea; 6 Department of Biomedical Sciences, Seoul National University Graduate School, Seoul, Republic of Korea; 7 Department of Biochemistry & Molecular Biology, Seoul National University College of Medicine, 103 Daehakro, Yeongun-dong, Jongno-gu, Seoul, 03080 Republic of Korea; 8 Cancer Research Institute, Seoul National University College of Medicine, Seoul, Republic of Korea; 9 Department of Family Medicine, Seoul National University Hospital, Seoul National University College of Medicine, 103 Daehakro, Yeongun-dong, Jongno-gu, Seoul, 03080 Republic of Korea

**Keywords:** Thyroid-stimulating hormone, Particulate matter, Genome-wide interaction study, Genome-wide association study, Functional association

## Abstract

**Background:**

Although the associations between air pollution exposure and thyroid function have been reported, the interactive effects of the genes involved remain unknown. Therefore, we aimed to identify candidate genetic loci involved in thyroid function by interacting with annual average exposure to particulate matter with a diameter of 10 microns or smaller (PM_10_) in Korean adults. A total of 1,863 and 1,458 adults were included in the discovery and replication steps, respectively. The average annual concentration of PM_10_ exposure was considered, and the participants were classified into two groups (low-to-moderate exposure and high-exposure groups) for binary analyses. A genome-wide single-nucleotide polymorphism analysis using a PM_10_ exposure interaction study was performed to determine thyroid-stimulating hormone levels in Korean adults.

**Results:**

Although no SNPs surpassed the stringent genome-wide significance threshold of *P*_int_ < 5E-08, one SNP (rs11781213) near *MSRA* reached a suggestive level of significance of *P*_int_ < 5E-07. Two genetic susceptibility loci (*FAM84B*/*PCAT1* and *STARD13*) were replicated at a nominal significance level of *P*_*int*_ < 1E-05 for the discovery cohort, and *P*_*int*_ < 0.05 for the replication cohort. A genetic variant (rs7169081 G > A) between *CGNL1* and *GCOM1* was of functional interest.

**Conclusions:**

This is the first study reporting genome-wide-air pollution interaction results for thyroid function. The association between long-term PM_10_ exposure and thyroid hormone levels may be partly explained by identifying several suggestive loci, including *MSRA*, *PCAT1*, and *GCOM1*.

**Supplementary Information:**

The online version contains supplementary material available at 10.1186/s41021-026-00357-z.

## Introduction

Short- and long-term exposure to air pollution is associated with an increased risk of various human diseases, including asthma, chronic obstructive pulmonary disease, stroke, certain types of cancer, and neurological and psychiatric disorders [1]. Health conditions caused by air pollution may lead to premature mortality [2]. A 2016 report from the Organization for Economic Cooperation and Development projected that premature deaths due to ambient air pollution would increase globally from 3 million in 2010 to 9 million in 2060. Therefore, health problems related to outdoor air pollution remain major global concerns.

Environmental toxicants, including air pollutants, negatively affect thyroid function [3–8]. Several studies have reported significant associations between air pollution exposure and changes in maternal and fetal thyroid hormone levels during pregnancy [3, 5, 7, 8]. The relationship between ambient air pollution and thyroid function appears stronger in obese individuals than in non-obese individuals [9, 10]. Certain populations may be more vulnerable to air pollution-induced thyroid dysfunction due to clinical or genetic factors. Genetic variations related to obesity may influence thyroid hormone levels through interactions with air pollution. Thyroid function is a complex trait shaped by both environmental and genetic components. Even at the same level of air pollution exposure, individuals show variability in thyroid, which may be partly explained by genetic susceptibility and gene–environment interactions.

Over the past decades, gene-by-air pollution interaction studies have mainly focused on specific genes (e.g., *GSTM1*, *GSTT1*, and *GSTP1*) selected based on biological plausibility, such as involvement in oxidative stress responses [11–13]. More recently, research has shifted toward genome-wide interaction approaches that test interactions across the entire genome rather than targeting only candidate genes [14–18]. This approach may help identify novel biological or genetic mechanisms beyond those already known to underlie the relationship between air pollution and thyroid hormone levels. However, interactive effects of air pollution exposure on thyroid function have not yet been reported at either the candidate gene or genome-wide level.

Therefore, we performed a genome-wide interaction study (GWIS) of annual particulate matter exposure (PM_10_; aerodynamic diameter ≤ 10 μm) in relation to thyroid-stimulating hormone (TSH) levels in Korean adults and identified several suggestive genetic loci.

## Methods

### Study population of the discovery and replication cohorts

This Seoul National University Hospital (SNUH) Healthcare Promotion Center (HPC) PM_10_ cross-sectional investigation was conducted to assess the association between genetic variations and various health indicators, including baseline anthropometric measurements and serum blood test results. Participants were recruited from the Health Promotion Center Main Branch (HPC1) and Healthcare System Gangnam Center (HPC2) at SNUH between December 2009 and December 2013.

For the discovery cohort, 2,102 individuals were initially enrolled, and 239 were excluded owing to missing information, including thyroid hormone–related phenotypes, invalid DNA samples, and lack of zip code data required to estimate exposure to ambient PM_10_ concentrations. Furthermore, individuals receiving thyroid hormone supplementation (Synthyroid) or antithyroid medication for underlying hypothyroidism or hyperthyroidism were excluded to minimize potential bias. Consequently, 1,863 adult males were included in the genetic analysis.

The replication cohort was recruited between 2014 and 2015 at the same hospital (HPC3). This cohort included 1,458 individuals, with genotyping and phenotypic data collected using procedures similar to those of the discovery cohort. The replication cohort was drawn from the main branch of SNUH, which served as the primary examination center.

This study was approved by the Institutional Review Boards of the SNUH Biomedical Research Institute and the National Cancer Center. Written informed consent was obtained from all participants. All study protocols and clinical procedures were conducted in accordance with the Declaration of Helsinki.

### Ascertainment of exposure to PM_10_ and outcome variable of TSH

Daily measurements of air pollutants (PM_10_) for approximately 300 monitoring stations across South Korea were collected from January 1, 2009 to December 31, 2015 (data obtained from www.airkorea.or.kr). The annual mean PM_10_ concentration for the year of examination was estimated for each participant based on their residential postal code. The nearest monitoring station to the participant’s residential address was selected to extrapolate annual PM_10_ exposure.

One-year average long-term exposure of PM_10_, calculated using the date of the healthcare examination and residential zip codes, was analyzed both as a continuous and a binary variable. For binary analyses, exposure was categorized using a cutoff of 53.5 µg/m^3^, corresponding to the 75th percentile of PM_10_ levels in the discovery cohort.

The main outcome variable, TSH, was measured from serum venous blood samples collected in the morning after at least 12 h of fasting, along with other blood tests such as total cholesterol, low-density lipoprotein cholesterol, and liver function enzymes. To approximate a normal distribution for GWIS analysis, the square root-transformed TSH value (TSHsr) was calculated (Kolmogorov–Smirnov *P* > 0.05).

### SNP genotyping and imputation

Genomic DNA was extracted from peripheral blood leukocytes of all participants using a QuickGene-610 L device (Fujifilm, Tokyo, Japan) following standard procedures. Samples in the discovery cohort were genotyped using the Human Core BeadChip kit (Illumina, San Diego, CA), whereas the replication cohort was genotypes using the Axiom Korean Chip version 1.1.

Genotype imputation was performed using the Northeast Asian Reference Database 2 (NARD2) whole-genome sequencing imputation panel [19]. To ensure data quality, SNPs were excluded if they failed Hardy–Weinberg equilibrium (*P* < 0.0005), had call rates below 99%, or had minor allele frequencies < 1%. After quality control, 4,677,241 SNPs were included in the final analysis.

### Functional expression mechanisms of potential enhancer activities

Enhancer activities associated with gene expression levels or gene–enhancer interactions were evaluated using experimentally validated activity-by-contact (ABC) models [20]. These models were annotated using the Cis-Element Atlas (http://catlas.org/humanenhancer/) across 220 different cell types [21].

Open chromatin regions relevant to gene expression in specific cell types were identified using single-cell Assay for Transposase-Accessible Chromatin sequencing data from the Cis-Element Atlas platform (https://catlas.org/catlas/). Gene expression patterns in human tissues were further validated using the Genotype-Tissue Expression (GTEx) [22] and Human Protein Atlas databases.

### Statistical analyses

A genome-wide association study (GWAS) of TSH in the discovery cohort was initially performed with age, examination center, and BMI as covariates, consistent with a previous TSH GWAS study [24]. A GWIS of TSH and PM_10_ was performed using PLINK version 1.9 (https://www.cog-genomics.org/plink/) with genotype data imputed using the NARD2 panel and stored in binary PLINK files (.bed,.bim, and.fam). During the GWIS, the “interaction” option with parameters specified for linear regression was applied to calculate interaction *P*-values between TSH and PM_10_ for each genotyped and imputed SNP. For the discovery set, the GWIS model formulae were as the follows:

For PM_10_ continuous analyses: Y(TSHsr) = 𝛽_0_ + 𝛽_1_ × age + 𝛽_2_ × center + 𝛽_3_ × BMI + 𝛽_4_ × SNP_ADD_ + 𝛽_5_ × PM_10_ + 𝛽_6_ × SNP_ADD_ × PM_10_.

For PM_10_ binary analyses: Y(TSHsr) = 𝛽_0_ + 𝛽_1_ × age + 𝛽_2_ × center + 𝛽_3_ × BMI + 𝛽_4_ × SNP_ADD_ + 𝛽_5_ × PM_10_b + 𝛽_6_ × SNP_ADD_ × PM_10_b.

Additive models were implemented for SNP effects, and *P*-values for 𝛽_6_ were extracted for GWIS analyses.

Manhattan plots and quantile–quantile(Q–Q) plots were generated using the R package “qqman” under both continuous and binary PM_10_ models. ANNOVAR [23] was used for functional annotation of SNPs, including intronic or exonic variants. For intergenic SNPs, the distance to each gene was calculated based on the hg19 reference genome. Beta coefficients and 95% confidence intervals from linear regression models were scaled to reflect a 1 µg/m^3^ increase in PM_10_.

The interaction significance threshold was set at a suggestive *P* < 1 × 10⁻⁵ for the discovery cohort and a nominal *P* < 0.05 for the replication cohort. The examination center (HPC1 and HPC2), age, and BMI were included as covariates in the discovery cohort, whereas age, sex, and BMI were included as covariates in the replication cohort.

All genotypic analyses were performed on a computing server at the Genomic Medicine Institute Research Service Center. Regional plots were created using LocusZoom (http://locuszoom.sph.umich.edu/locuszoom/). STATA version 17 (StataCorp, LLC, TX, USA) was used for adjusted linear regression, interaction, and stratified association analyses.

## Results

### Baseline demographics of the study population

The mean age ± standard deviation (SD) of the discovery cohort (HPC1 and HPC2) was 48.9 ± 7.0 years (range: 21–68 years), compared with 54.4 ± 11.8 years in the replication cohort. The discovery cohort consisted solely of men, whereas 58% of participants in the replication cohort were men.

The mean BMI was 24.7 ± 2.8 kg/m^2^ in the discovery cohort and 23.6 ± 3.1 kg/m^2^ in the replication cohort. The mean TSH levels were 1.9 mIU/L and 1.8 mIU/L, respectively, and the mean PM_10_ concentrations were 49.3 µg/m^3^ and 48.0 µg/m^3^ (Table 1).

Within the discovery cohort, participants in the 1st to 3rd quartiles of PM_10_ (*N* = 1398) were allocated to the low-to-moderate exposure group (27.1–53.5 µg/m^3^), whereas those in the 4th quartile (*N* = 465) were assigned to the high-exposure group (53.5–82.6 µg/m^3^).

**Table 1:** Baseline characteristics of the SNUH Health Promotion Center (HPC) cohort according to PM_10_ exposure and TSH levels

Characteristics	Discovery Step		Replication Step
	*N* (%) or mean (SD)		*N* (%) or mean (SD)
Year of sample recruitment	2009–2013		2014–2015
Site	HPC1	HPC2	HPC3
N	1355	508	1458
Age (years)	49.1 (7.1)	48.5 (6.5)	54.4 (11.8)
Sex			
Men	1355 (100%)	508 (100%)	847 (58.1%)
Women	-	-	611 (41.9%)
BMI (kg/m^2^)	24.6 (2.78)	24.7 (2.77)	23.6 (3.1)
Thyroid hormones			
Free T_4_ (ng/dL)	1.36 (0.41)	1.35 (0.23)	1.30 (0.26)
TSH (mIU/L)	1.94 (1.78)	1.74 (0.95)	1.81 (2.04)
PM_10_ (µg/m^3^)			
Mean	48.0 (7.2)	52.8 (6.7)	48.0 (6.0)
25th percentile	42.9	48.9	44.7
50th percentile (median)	47.4	52.4	46.9
75th percentile	52.2	54.7	51.0

BMI, body mass index; PM, particulate matter; SD, standard deviation

### GWAS of TSH in the discovery cohort and replication using previously published TSH GWAS

A GWAS of TSH levels was performed in the discovery cohort (HPC1 and HPC2 combined), treating TSH as a continuous variable. The square root of TSH was used to approximate a normal distribution. Age, examination center, and BMI were included as covariates (*N* = 1,931 participants with non-missing data). Genome-wide significance was defined as *P* < 5 × 10^− 8^, and suggestive significance was set at *P* < 1 × 10^− 5^ for the top signals at each locus. The genomic inflation factor was 0.997.

**Fig. 1:**
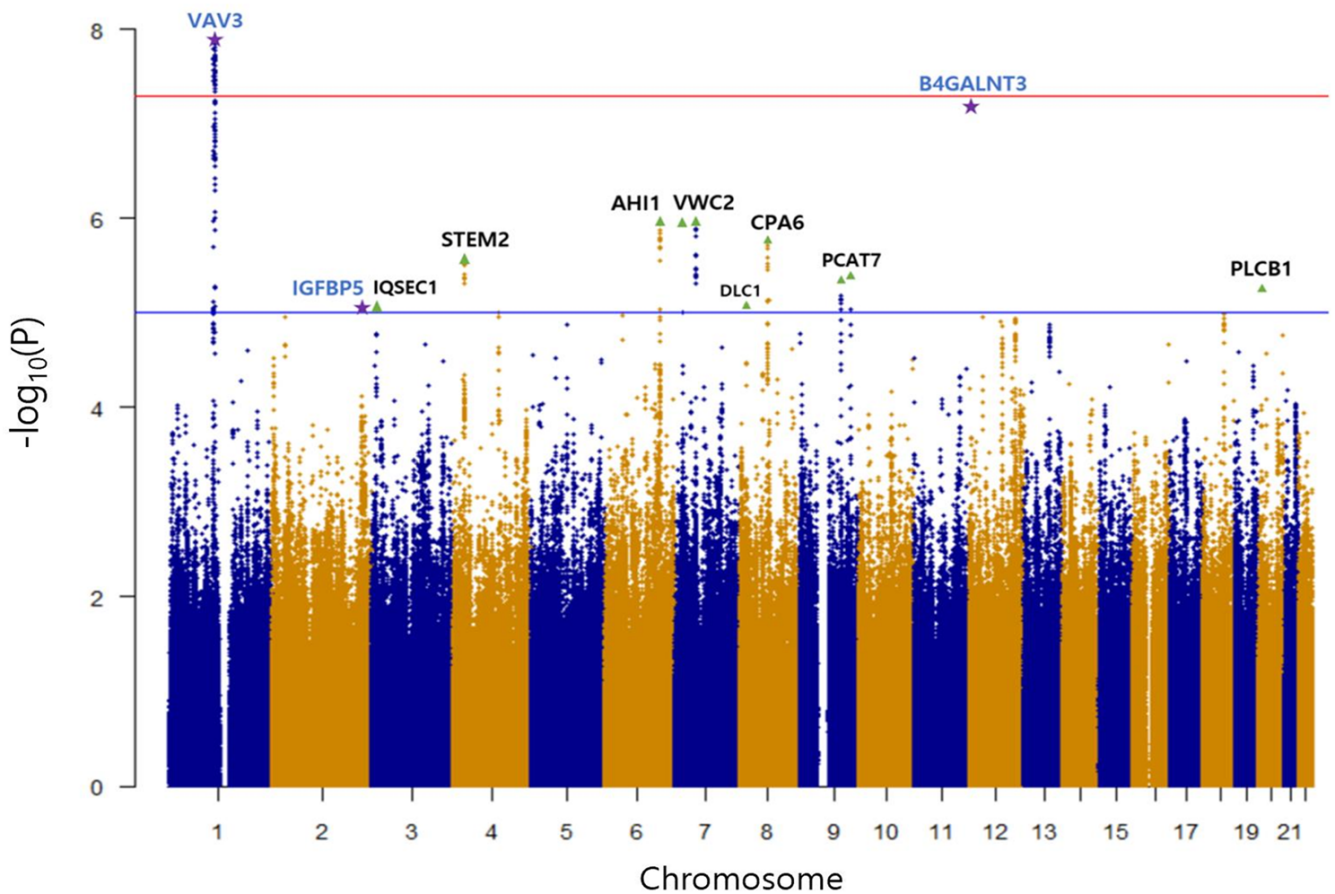
Manhattan plot of the GWAS of TSH levels in the discovery cohort (HPC1 and HPC2). The x-axis indicates genomic position across chromosomes, and the y-axis shows the -log_10_ of GWAS *P*-values. Age, examination center, and body mass index were included as covariates. Replicated signals from the previously published GWAS are indicated by purple stars. The red line denotes the genome-wide significance threshold (*P* = 5E-08), and the blue line denotes the suggestive significance threshold (*P* = 1E-05)

Three top loci were replicated in accordance with a previously published GWAS of TSH levels [24] (Fig. 1; replicated signals marked with purple stars). The only locus that reached genome-wide significance was *VAV3* on chromosome 1, with a lead signal *P* = 1.31E-08. This gene was also statistically significant in previous TSH GWAS [24], which reported associations in 343,604 individuals, primarily of European ancestry. Replication was observed not only at the gene level but also at the SNP level, as rs78499451 (*P* = 3.83E-08) was also significant in the published study.

Several loci reached suggestive significance (marked with green triangles). Among the top signals, additional replicated loci included *IGFBP5* on chromosome 2 (*P* = 9.08E-06) and *B4GALNT3* on chromosome 12 (*P* = 6.25E-08). *IGFBP5* has previously been implicated in the regulation of thyroid-relevant pathways. All three loci (*VAV3*, *IGFBP5*, and *B4GALNT3*) were associated with hypothyroidism but not hyperthyroidism in the prior GWAS study [24].

### GWIS of PM_10_ and TSH in the discovery and replication cohorts

GWIS results were examined under two exposure models, in which PM_10_ was analyzed as either a continuous or a binary variable (cutoff value: 53.5 µg/m^3^).

In the continuous analysis, rs6998646 near *PCAT1* and two SNPs in high linkage disequilibrium (LD) (rs79013868 and rs9597585; *r*^2^ = 0.75) located within an intron of *STARD13* showed statistical significance in both the discovery and replication cohorts.

**Fig. 2:**
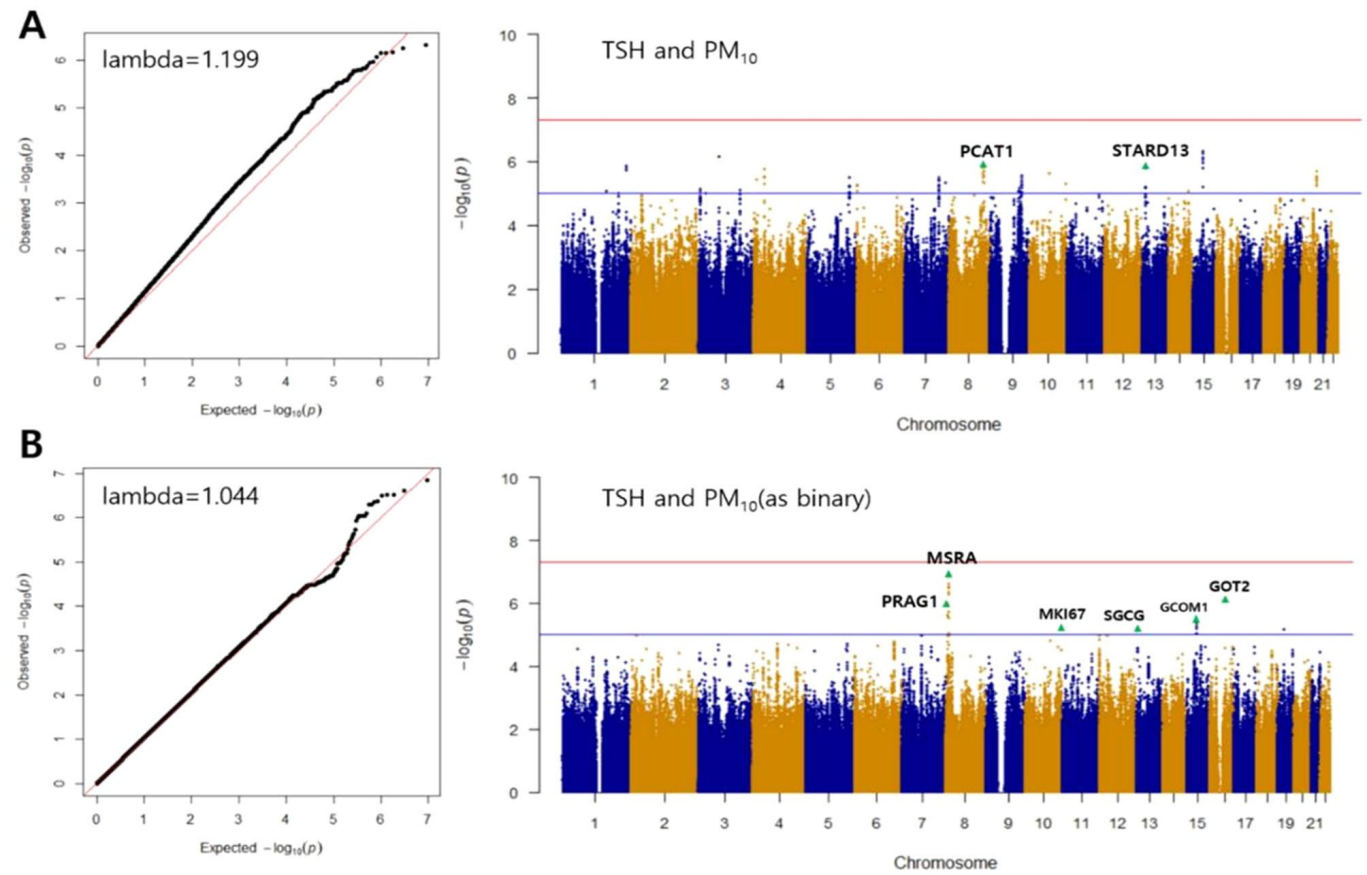
Quantile–quantile (Q–Q) and Manhattan plots for the GWIS of (**A**) TSH-PM_10_ (continuous) and (**B**) TSH-PM_10_ (binary). Q–Q plots illustrate the extent of genomic inflation in the dataset. Deviation from the expected distribution (y = x) is quantified using the genomic inflation factor (lambda)

In the binary analysis, seven SNPs (representing the lead SNPs at each locus in the Manhattan plot) reached statistical significance in the discovery cohort but were not replicated (Table 2; Fig. 2). Among these, two SNPs (rs11781213 and rs7837316) were located near or within *MSRA*. The remaining SNPs were located near *GOT2*, *PRAG1*, *GCOM1*, *MKI67*, and *SGCG* (rs73563822, rs10109092, rs7169081, rs879526736, and rs9510483, respectively).

Only rs879526736 was directly genotyped on the microarray platform, whereas the other SNPs listed in Table 2 were imputed using the NARD2 reference panel. Q–Q and Manhattan plots indicated that genomic inflation factors were within an acceptable range (< 1.20) for both analyses (Fig. 2). The SNPs with the lowest *P*_int_ values in Table 2 were also labeled with gene names in green triangles in the Manhattan plot (Fig. 2**)**.

**Fig. 3:**
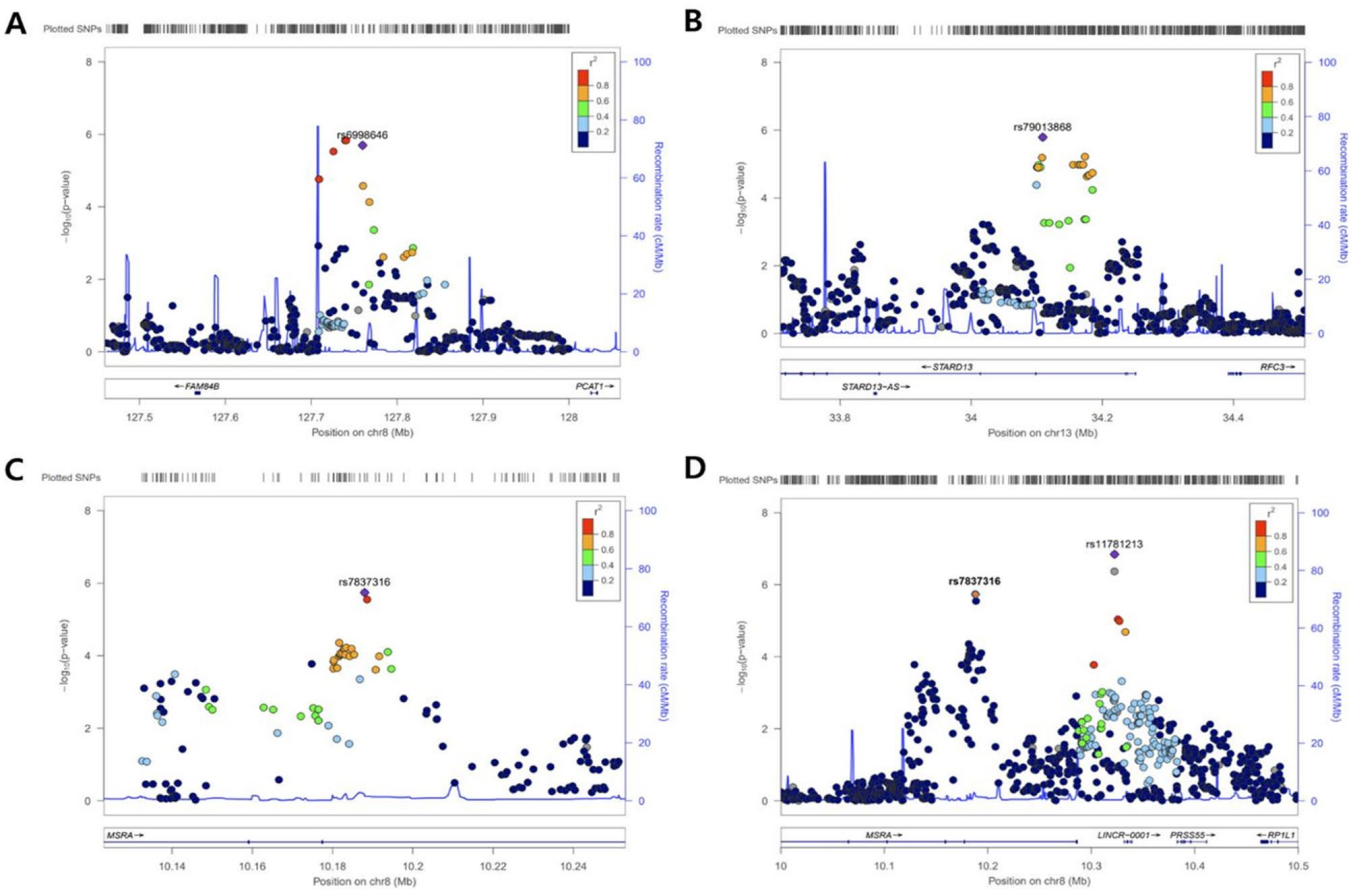
Regional association plots for SNPs near (**A**) rs6998646 (*FAM84B; PCAT1*) and (**B**) rs79013868 (*STARD13*) loci based on PM_10_ interaction, and (**C**) rs7837316 (*MSRA*) and (**D**) rs11781213 (*MSRA; LINCR-0001*) based on PM_10_ (binary) interaction

LocusZoom plots were generated for the peak loci (*PCAT1*, *STARD13*, and *MSRA*) to illustrate LD patterns within each cluster **(**Fig. 3**)**. For *MSRA*, two main peak SNPs were observed: the intronic variant rs7837316 and the intergenic variant rs11781213.

**Table 2:** Genome-wide interaction study results of PM_10_ (continuous and binary) for TSH

Trait	Chr	SNP	Position^a^	Major/Minor allele	MAF	Type	Nearest genes (distance, kb)	Discovery set (*N* = 1,863)		Replication set (*n* = 1,458)		*P*_combined_
								β (SE)	*P*_int_	β (SE)	*P*_int_	
TSH and PM_10_												
	8	rs6998646	127,759,740	T/G	0.217	Intergenic	*FAM84B; PCAT1* (189; 266)	0.010 (0.002)	2.01E-06	0.010 (0.003)	0.003	**7.75E-09**
	13	rs79013868^b^	34,109,066	G/A	0.113	Intron	*STARD13*	-0.014 (0.003)	1.62E-06	-0.011 (0.005)	0.017	**3.74E-07**
	13	rs9597585^b^	34,108,026	A/T	0.124	Intron	*STARD13*	-0.013 (0.003)	6.48E-06	-0.008 (0.004)	0.049	2.45E-06
TSH and PM_10_(as binary)												
	8	rs11781213	10,322,546	C/T	0.456	Intergenic	*MSRA; LINCR-0001* (36;10)	-0.163 (0.031)	**1.42E-07**	-0.019 (0.042)	0.661	3.45E-05
	16	rs73563822	59,410,868	T/C	0.176	Intergenic	*GOT2;APOOP5* (643;377)	0.198 (0.040)	8.91E-07	0.035 (0.059)	0.550	3.02E-05
	8	rs10109092	8,383,660	T/C	0.435	Intergenic	*PRAG1;CLDN23* (140;176)	0.157 (0.032)	9.70E-07	0.062 (0.047)	0.188	5.10E-06
	8	rs7837316	10,187,992	G/A	0.335	Intron	*MSRA*	-0.162 (0.034)	1.82E-06	-0.015 (0.046)	0.739	1.17E-04
	15	rs7169081	57,845,795	G/A	0.492	Intergenic	*CGNL1;GCOM1* (3;38)	0.145 (0.031)	3.37E-06	0.044 (0.042)	0.304	1.25E-05
	10	rs879526736^c^	130,060,346	G/A	0.167	Intergenic	*MKI67;LINC01163* (136;24)	0.183 (0.041)	6.70E-06	-0.091 (0.061)	0.135	8.31E-03
	13	rs9510483	23,555,339	C/T	0.175	Intergenic	*LINC00621;SGCG* (65;179)	0.185 (0.041)	7.39E-06	0.046 (0.058)	0.434	4.09E-05

^a^SNP positions are based on human GRCh37/hg19 from UCSC Genome Browser

^b^These SNPs are in the strong linkage disequilibrium (LD) relationship (r^2^_LD_ = 0.75; D´ = 0.91)

^c^rs879526736 is the only SNP genotyped, and the rest are imputed using the NARD panel v.2. Bold case means a *P*_int_ of below 5E-07

### Interaction between PM_10_ and TSH levels in continuous and binary analyses based on GWIS results

**Fig. 4:**
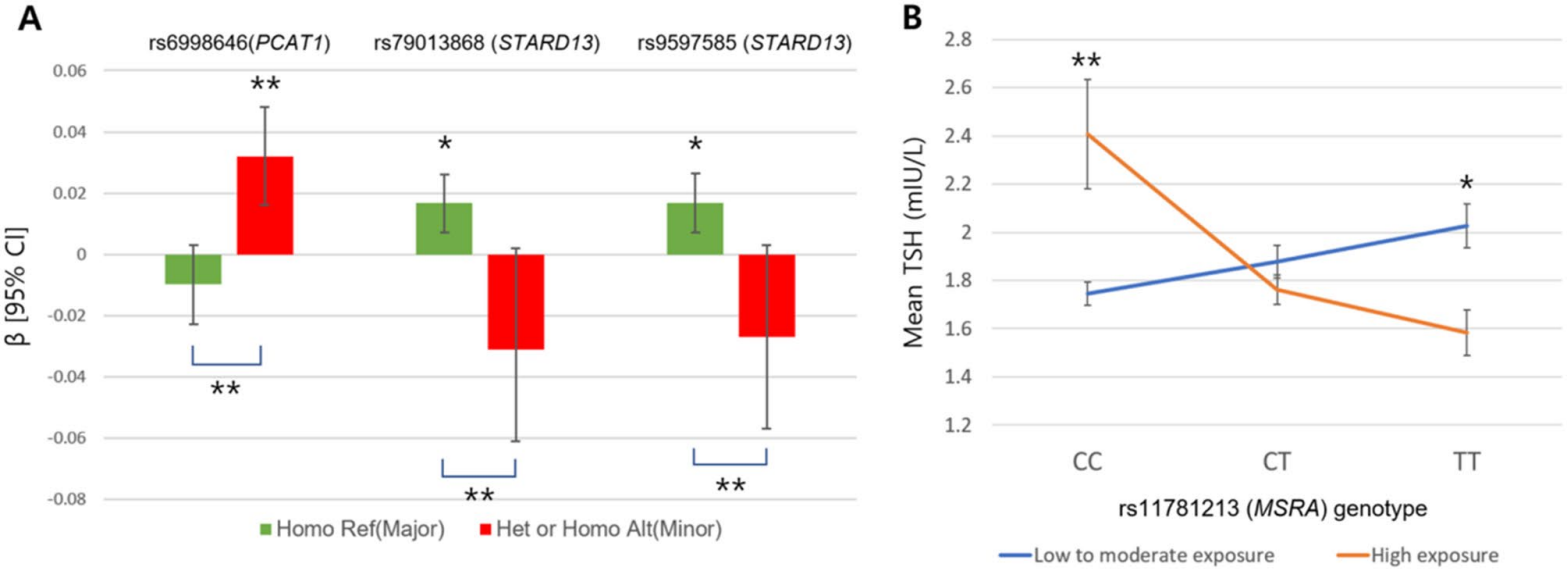
Beta coefficients and 95% confidence intervals for the interaction between PM_10_ exposure and SNP genotypes in linear regression models of TSH levels. (**A**) Replicated SNPs from the continuous PM_10_ analysis (**B**) Interaction between rs11781213 near *MSRA* and binary PM_10_ exposure in modulating TSH levels (*P < 0.05, **P < 0.001)

For the three replicated GWIS SNPs in the PM_10_ continuous analysis **(**Table 2**)**, the signs of the beta coefficients derived from TSH linear regressions models across PM_10_ exposure differed by genotype subgroup. Specifically, the direction of the association was reversed when comparing the reference allele homozygous group with the heterozygous or alternative allele homozygous group (Table 3; Fig. 4A). For rs6998646 (*FAM84B; PCAT1*), the beta value changed from − 0.010 to 0.032. For rs79013868 (*STARD13*), the beta direction changed from 0.017 to -0.031, and for rs9597585 (*STARD13*), the beta value changed from 0.017 to -0.027. All comparisons of these signs reversals were statistically significant.

For the top *P*_int_ SNP in the PM_10_ binary variable analysis, TSH levels differed significantly between the CC and TT genotypes of rs11781213 near *MSRA* when stratified by low-to-moderate versus high PM_10_ exposure. The slope of the association with TSH was reversed between the exposure groups, supporting an interaction between this *MSRA* variant and PM_10_ levels on TSH (Fig. 4B).

**Table 3:** Stratified association results between PM_10_ exposure as a continuous variable and TSH according to the genotypes of rs6998646 (*FAM84B; PCAT1*) and rs79013868/rs9597585 (*STARD13*) SNPs within the discovery cohort

Traits	SNP	Major/Minor allele	Homo Ref (Major)			Het or Homo Alt (Minor)		
			*N*	β (95% CI)	*P*_assoc_	*N*	β (95% CI)	*P*_assoc_
TSH and PM_10_								
	rs6998646 *(FAM84B; PCAT1)*	T/*G*	1146	-0.010 (-0.023, 0.004)	0.147	717	0.032 (0.016, 0.048)	6.57E-05
	rs79013868 *(STARD13)*	G/*A*	1467	0.017 (0.007, 0.026)	0.001	396	-0.031 (-0.064, 0.002)	0.063
	rs9597585 *(STARD13)*	A/*G*	1429	0.017 (0.007, 0.027)	0.001	434	-0.027 (-0.057, 0.002)	0.071

TSH, thyroid-stimulating hormone; SNP, single-nucleotide polymorphism; N, number of participants; CI, confidence interval; Assoc, association; Ref, reference allele; Alt, alternative allele

The beta coefficients and 95% confidence intervals are scaled to the 1 µg/m^3^ increase in PM_10_.

The association results were adjusted for recruitment site, age, and body mass index

The effect allele of each SNP is underlined within the major and minor alleles

### ABC model of rs7169081 as a potential enhancer of *GCOM1*

The GWIS of PM_10_ exposure and TSH levels identified rs7169081 G > A, an intergenic SNP located between *CGNL1* and *GCOM1* at locus 15q21.3, as showing a significant interaction at the cutoff of *P*_int_ < 1E-05. The A allele was associated with increased TSH levels following PM_10_ exposure.

This SNP lies within enhancer regions linked to *GCOM1* and *CGNL1*, as indicated by the ABC model and single-cell Assay for Transposase-Accessible Chromatin data, with enhancer activity observed mainly in atrial cardiomyocytes. Elevated *GCOM1* expression was also primarily observed in cardiomyocytes according to the GTEx and Human Protein Atlas databases.

**Fig. 5:**
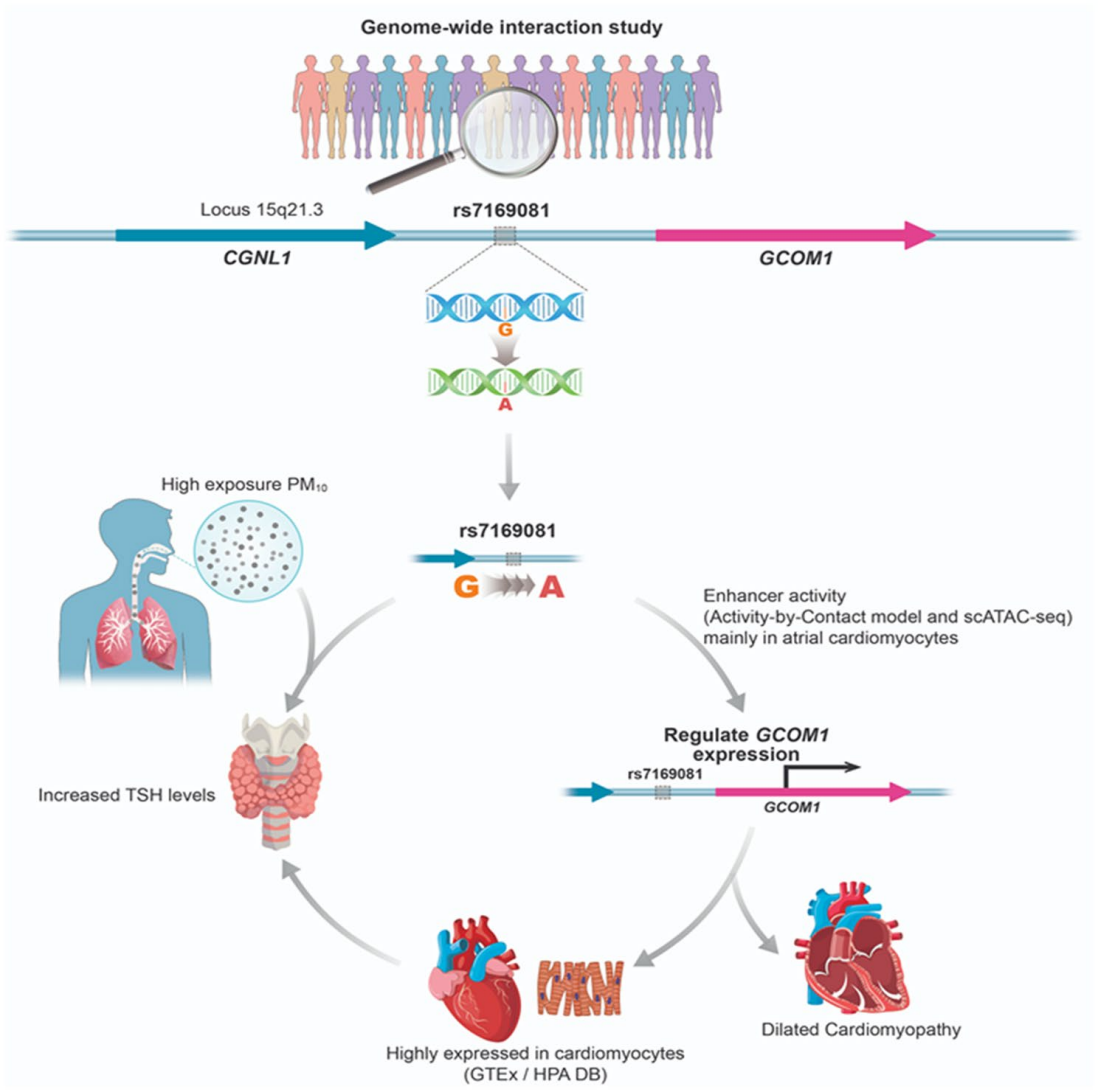
Functional validation of rs7169081 located between *CGNL1* and *GCOM1*

*GCOM1* has been implicated in dilated cardiomyopathy [25], and cardiomyocytes are known to be influenced by thyroid hormones [26], consistent with the GWIS findings reported here. Furthermore, rs7169081 was associated with thyroid expression quantitative trait loci (eQTL) signals related to *CGNL1* in the GTEx database (*P* = 7.50E-06), suggesting a potential link with TSH regulation. This variant was also significant as a whole-blood eQTL enhancing *GCOM1* expression (*P* = 1.10E-13) (Fig. 5).

## Discussion

A genome-wide SNP-by-PM_10_ exposure interaction study on TSH levels was conducted in Korean adults. One SNP (rs11781213) near *MSRA* reached a suggestive significance level (*P*_int_ < 5E-07) in the binary PM_10_ analysis of the discovery cohort. In addition, three SNPs identified in the continuous PM_10_ analysis were replicated in the replication cohort after meeting the discovery threshold of *P*_int_ < 1E-05. These SNPs, rs6998646 (*FAM84B; PCAT1*), rs79013868 (*STARD13*), and rs9597585 (*STARD13*), showed significant interactions in the replication cohort at a nominal level of *P*_*int*_ < 0.05. Rs6998646 is an intergenic SNP between *FAM84B* and *PCAT1* and was associated with increased TSH levels through interaction with PM_10_. The other two SNPs (rs79013868 and rs9597585) were intronic variants within *STARD13* and were in high LD (r^2^_LD_ = 0.75). The lead SNP in the binary PM_10_ analysis (rs11781213 near *MSRA*) was not significant in the replication cohort (*P*_int_ = 0.661). A genetic variant (rs7169081 G > A), located between *CGNL1* and *GCOM1* on chromosome 15, showed enhancer-related activity in atrial cardiomyocytes in the ABC model.

Furthermore, a GWAS of TSH levels alone in the discovery cohort supported the internal validity of the dataset, as one genome-wide significant locus (*VAV3*) and two suggestive loci (*IGFBP5* and *B4GALNT3*) were consistent with findings from a previous larger European GWAS of TSH.

*FAM84B* (also known as *LRATD2*) contributes to the progression of various cancers and promotes breast cancer tumorigenesis through NF-kB activation [27]. NF-kB signaling regulates immune and inflammatory responses [28] and has been implicated in thyroid autoimmunity and thyroid carcinogenesis [29]. In contrast, *PCAT1* overexpression inhibits ferroptosis [30], a process involving iron metabolism and lipid peroxidation [31], which may influence thyroid hormone regulation [32]. Notably, exposure to fine particulate matter (PM_2.5_) increases ferroptosis sensitivity in human endothelial cells [33]. Thus, synergistic effects between air pollution exposure and genetic variation in *PCAT1* may affect thyroid function through enhanced susceptibility to ferroptosis.

*STARD13* is a tumor suppressor, and its downregulation is associated with poor prognosis in papillary thyroid carcinoma [34]. However, the mechanisms linking these genes to air pollution remain unclear. Another SNP (rs73563822) near *GOT2* was identified in the binary PM_10_ GWIS. The thyroid hormone receptor complex regulates fatty acid-binding proteins including GOT2 [35]. Additionally, rs10109092 near *PRAG1* was identified in this study, and the *PRAG1* locus has also been reported in a GWAS of TSH [36], suggesting a broader role of TSH-associated polygenic variation in thyroid cancer risk.

Air pollution may have a stronger relationship with thyroid hormone levels in certain susceptible subgroups. Associations between PM exposure and TSH appear more pronounced in overweight or obese individuals [10], particular among those with high visceral adiposity [9]. *MSRA* encodes methionine sulfoxide reductase A, which reduces methionine sulfoxide to methionine and is expressed across multiple tissues, including liver, kidney, and brain [37]. Variants in *MSRA* have been linked to abdominal and early-onset extreme obesity [38–40], and reduced *MSRA* activity has been observed in obese rat models with increased visceral adiposity [41].

*MSRA* plays an important role in oxidative stress and inflammatory regulation, providing a potential mechanistic link between air pollution exposure and thyroid hormone dysregulation. Mice lacking *MSRA* (*MSRA* -/-) are more sensitive to oxidative stress than wild-type controls [42]. Furthermore, *MSRA* silencing stimulates inflammatory responses by upregulating pro-inflammatory cytokines such as *TNF*-*α* and *IL-1β* and increasing reactive oxygen species levels [43]. Collectively, the interaction between *MSRA* variants and PM_10_ exposure identified in this study supports previously described links among air pollution, abdominal obesity, and thyroid function.

This study also highlighted rs7169081 near *GCOM1*, within the GRINL1A complex transcription unit, which is highly expressed in cardiomyocytes [25]. This SNP was associated with increased thyroid tissue expression based on *CGNL1* eQTL data of GTEx [22]. Thyroid hormones modulate cardiomyocyte-specific target genes such as *MYH6* and *MYH7* and influence ion channel activity, including Na^+^/K^+^ and Ca^2+^ signaling pathways [26]. Triiodothyronine has been shown to affect cardiomyocyte morphology [44] and fetal cardiomyocyte maturation [45]. Therefore, this functional variant near *CGNL1* and *GCOM1* may influence biological pathways in both cardiomyocytes and thyroid tissues, thereby strengthening the relationship between PM_10_ exposure and TSH regulation.

Gene–air pollution interactions in human diseases have historically focused on candidate genes involved in oxidative stress and systemic inflammation. This is the first genome-wide gene-by-air pollution interaction analysis of thyroid hormones regulation. Genotypic imputation using an Asian-based reference panel [19] minimized loss of genetic information. These suggestive loci identified (*PCAT1*,* STARD13*,* MSRA*, and *GCOM1*) may improve understanding of genetic susceptibility to air pollution effects on thyroid function, particularly in Asian populations.

Despite its strengths, this study has limitations. Individual exposure was estimated based only on residential zip code, owing to the lack of information on indoor exposure or workplace location. PM_2.5_ exposure could not be included because national monitoring data were not available until 2015. Furthermore, no SNP surpassed the stringent genome-wide significance limit of 5E-08, which may be overly conservative for gene–environment interaction analyses. Therefore, more relaxed thresholds were applied (*P*_int_ < 5E-07 for combined interaction signals, *P*_int_ < 1E-05 discovery and *P*_int_ < 0.05 in replication). Finally, the cross-sectional design precludes causal inference between particulate matter exposure and altered TSH levels across genotypes.

In conclusion, this study investigated genome-by-PM_10_ exposure interactions related to thyroid function in Korean adults and identified several plausible genetic loci. The biological and pathological roles of these candidate genes support potential synergistic effects of PM_10_ exposure on thyroid hormone regulation. Further studies in other ethnic groups are required to validate these interactions and clarify underlying mechanisms. These findings highlight the importance of genome-wide approaches for understanding novel genetic modifiers of air pollution effects on thyroid function.

## Supplementary Information

Below is the link to the electronic supplementary material.

Supplementary Material 1

## Data Availability

All data used and/or analyzed in the current study are available from the corresponding author upon reasonable request.
