# Supplementary material for: A genome-wide interaction study of thyroid-stimulating hormone levels and particulate matter exposure among Koreans
Source: Genes Environ. 2026 Mar 5;48:10. doi: 10.1186/s41021-026-00357-z (PMC13041225; doi:10.1186/s41021-026-00357-z)
Supplement: Supplementary file 1 — Supplementary Material 1 [file 41021_2026_357_MOESM1_ESM.docx]

**Supplementary Information**

**A genome-wide interaction study of thyroid-stimulating hormone levels and particulate matter exposure among Koreans**

**1. Plink Linux statistical codes for GWIS analysis**

1) Continuous PM_10_ analysis in the discovery cohort:

./plink --allow-no-sex --bfile SNUHFM_NARD2_imputation --ci 0.95 --covar SNUHFM_pheno.txt --covar-name center,age,bmi,PM10 --hwe 0.0005 --linear interaction --maf 0.1 --out output --parameters 1,2,3,4,5,9 --pheno SNUHFM_pheno.txt --pheno-name TSHsr

2) Binary PM_10_ analysis in the discovery cohort:

./plink --allow-no-sex --bfile SNUHFM_NARD2_imputation --ci 0.95 --covar SNUHFM_pheno.txt --covar-name center,age,bmi,PM10_b --hwe 0.0005 --linear interaction --maf 0.1 --out output --parameters 1,2,3,4,5,9 --pheno SNUHFM_pheno.txt --pheno-name TSHsr

3) Continuous PM_10_ analysis in the replication cohort:

./plink --allow-no-sex --bfile SNUHFM_NARD2_imputation --ci 0.95 --covar SNUHFM_pheno.txt --covar-name age,sex,bmi,PM10 --hwe 0.0005 --linear interaction --maf 0.1 --out output --parameters 1,2,3,4,5,9 --pheno SNUHFM_pheno.txt --pheno-name TSHsr

4) Binary PM_10_ analysis in the replication cohort:

./plink --allow-no-sex --bfile SNUHFM_NARD2_imputation --ci 0.95 --covar SNUHFM_pheno.txt --covar-name age,sex,bmi,PM10_b --hwe 0.0005 --linear interaction --maf 0.1 --out output --parameters 1,2,3,4,5,9 --pheno SNUHFM_pheno.txt --pheno-name TSHsr

# TSHsr: square-root of TSH, PM10_b: binary variable of PM_10_

# parameter option indicates SNP_ADD_, center, age, bmi, PM10, SNP_ADD_xPM_10_, respectively, where the interaction *P*-values for SNP_ADD_ x PM_10_ was further extracted from the output file.

**2. Full names for genes mentioned in this study (in alphabetical order)**

*APOOP5*: Apolipoprotein O Pseudogene 5

*B4GALNT3*: Beta-1,4-N-Acetyl-Galactosaminyltransferase 3

*CGNL1*: Cingulin Like 1

*CLDN23*: Claudin 23

*FAM84B*: Family with sequence similarity 84, member B

*GCOM1*: GRINL1A complex locus 1

*GOT2*: Glutamic-Oxaloacetic Transaminase 2

*GSTM1*: Glutathione S-Transferase Mu 1

*GSTP1*: Glutathione S-Transferase Pi 1

*GSTT1*: Glutathione S-Transferase Theta 1

*IGFBP5*: Insulin Like Growth Factor Binding Protein 5

*LINC, LINCR*: Long Intergenic Non-Protein Coding RNA

*LRATD2*: LRAT Domain Containing 2

*MKI67*: Marker of Proliferation Ki-67

*MSRA*: Methionine sulfoxide reductase A

*MYH*: Myosin Heavy Chain

*PCAT1*: Prostate cancer associated transcript 1

*PRAG1*: PEAK1 Related, Kinase-Activating Pseudokinase 1

*SGCG*: Sarcoglycan Gamma

*STARD13*: StAR-related lipid transfer domain containing 13

*VAV3*: Vav Guanine Nucleotide Exchange Factor 3
